# Auto‐segmentation of thoraco‐abdominal organs in pediatric dynamic MRI

**DOI:** 10.1002/mp.70104

**Published:** 2025-11-08

**Authors:** Yusuf Akhtar, Jayaram K. Udupa, Yubing Tong, Tiange Liu, Caiyun Wu, Rachel Kogan, Mostafa Al‐Noury, Mahdie Hosseini, Leihui Tong, Samarth Mannikeri, Dewey Odhner, Joseph M. Mcdonough, Carina Lott, Abigail Clark, Patrick J. Cahill, Jason B. Anari, Drew A. Torigian

**Affiliations:** ^1^ Medical Image Processing Group, Department of Radiology University of Pennsylvania Philadelphia Pennsylvania USA; ^2^ School of Computer Science Engineering and Information Systems Vellore Institute of Technology, Katpadi Vellore Tamil Nadu India; ^3^ School of Intelligence Science and Technology University of Science and Technology Beijing Beijing China; ^4^ Boston University College of Engineering Boston University Boston USA; ^5^ The Wyss/Campbell Center for Thoracic Insufficiency Syndrome Children's Hospital of Philadelphia Philadelphia Pennsylvania USA

**Keywords:** adolescent idiopathic scoliosis, deep neural networks, dynamic MRI, early onset scoliosis, image segmentation, thoracic insufficiency syndrome, thoraco‐abdominal organs

## Abstract

**Purpose:**

Dynamic magnetic resonance imaging (dMRI) is a practical imaging modality for capturing information about regional thoracic‐abdominal components and their dynamics in healthy children and pediatric patients with thoracic insufficiency syndrome (TIS). We propose an auto‐segmentation set‐up for the lungs, kidneys, liver, spleen, and thoraco‐abdominal skin outer boundary (Skn) in dMRI images.

**Methods:**

The segmentation setup has been implemented in two steps, recognition and delineation, using two deep neural network (DL) architectures, DL‐R and DL‐D for the recognition and delineation steps, respectively. The encoder‐decoder framework in DL‐D utilizes features at four different resolution levels to counter the challenges involved in segmentation. dMRI sagittal slice acquisitions of 189 (near‐)normal subjects were evaluated, with an in‐plane spatial resolution of roughly 1 × 1 mm^2^ with 6.00 mm spacing between slices. We utilized images from 89 and 10 subjects at end inspiration for training and validation, respectively. For testing, we experimented with three scenarios utilizing: (1) the images of the 90 (=189‐89‐10) remaining subjects at end inspiration for testing, (2) the images of the remaining 90 subjects at end expiration for testing, and (3) the images of the other 99 (=89+10) subjects at end expiration for testing. In some situations, we can take advantage of the already available ground truth (GT) segmentation for an object in a subject at a particular respiratory phase to automatically segment the same object in the same subject at a different respiratory phase, and then refine the segmentation to create the final GT for all respiratory phases in the image of a subject. We anticipate that this process of creating GT would require minimal post hoc correction. In this spirit, we conducted separate experiments where we assumed to have GT of test subjects at the end expiration for scenario (1), end inspiration for (2), and end inspiration for (3). A major contribution in this paper is the different scenarios of training and testing that we have extensively evaluated with respect to respiratory phases and the subjects to which the images in the training and testing sets belong.

**Results:**

Among these three scenarios of testing, for DL‐R, we achieve the best average location error (LE) of about 1 voxel for the lungs, kidneys, and spleen, and 1.5 voxels for the liver and Skn. The standard deviation (SD) of LE is about 1 or 2 voxels. For DL‐D, we achieve an average Dice coefficient (DC) of about 0.92 to 0.94 for the lungs, 0.82 for the kidneys, 0.90 for the liver, 0.81 for the spleen, and 0.93 for Skn. The SD of DC is lower (0.02 to 0.07) for the lungs, liver, and Skn and slightly higher (0.06 to 0.12) for the spleen and kidneys.

**Conclusions:**

Motivated by applications in surgical planning for disorders such as TIS, adolescent idiopathic scoliosis, and early onset scoliosis, we have created an auto‐segmentation system for thoraco‐abdominal organs in dMRI acquisitions. This proposed setup copes with the challenges posed by low resolution, motion blur, inadequate contrast, and image intensity non‐standardness in dMRI images quite well.

## 1 INTRODUCTION

The study of the dynamics of thoraco‐abdominal organs such as the lungs, liver, spleen, and kidneys is important in patients with respiratory restrictive disorders such as thoracic insufficiency syndrome (TIS)^1^ (a pediatric disorder in which there is inability of the thorax to support normal respiration or lung growth), adolescent idiopathic scoliosis (AIS),^2^, ^3^ and early onset scoliosis (EOS).^4^ For example, the dynamic motion parameters can be compared with those of near‐normal subjects to understand the deviation from normality of the architecture and motion of the organs in these patients. Such dynamic properties during respiration can be captured effectively via dynamic magnetic resonance imaging (dMRI),^1^ which does not involve radiation exposure, does not require special patient maneuvers or breathing control, and can be implemented readily on MRI scanners available in the community. In this paper, we focus on the problem of auto‐segmentation of the lungs, kidneys, liver, spleen, and thoraco‐abdominal skin outer boundary (collectively referred to as **O**) in dMRI sagittal acquisitions of healthy subjects, which is a necessary first step before carrying out motion analysis.

We identified related work using “dynamic MRI article,” “dynamic MRI segmentation article,” and “dynamic MRI thorax” in the Google search engine. Articles^5^, ^6^ were listed with “dynamic MRI article,” which presented cardiac motion and musculoskeletal joint motion, respectively, from a clinical perspective only. The search “dynamic MRI segmentation article” listed 5 articles,^7^, ^8^, ^9^, ^10^, ^11^ which were related to the segmentation of a single object of interest. For example, ^9^ dealt with segmentation of blood vessels, and ^11^ segmented the skin from axial slices of the breast. Three articles,^12^, ^13^, ^14^ which were listed with “dynamic MRI thorax,” utilized only manual segmentations of the diaphragm or chest wall excursions for measuring relevant physiological parameters. There exist two works^15^, ^16^ which deal with the segmentation of the lungs in dMRI images. To the best of our knowledge, methods dealing with multi‐organ (> 2) automatic segmentation in dMRI acquisitions, especially of the thorax/abdomen, do not exist. We will discuss the related works^15^, ^16^ and other articles referenced in this paragraph in further detail in the next section.

**FIGURE 1:**
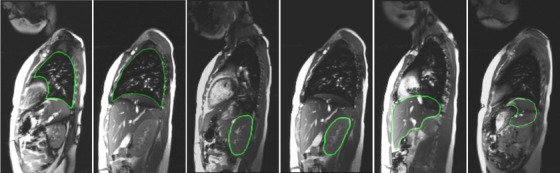
Representative sagittal bright‐blood dMRI slices at end‐expiratory phase (obtained with 4D construction^17^ from a dMRI acquisition) through the thorax and abdomen of a normal subject with true boundary delineations for (left to right) left lung, right lung, left kidney, right kidney, liver, and spleen.

Dynamic MRI images inherit the problems of static MRI images and are more severe such as (1) different meaning of gray‐level intensities for the same object for the same subject across different acquisitions, and for the same object across different subjects, (2) poor contrast among objects, (3) low signal‐to‐noise ratio, (4) motion blur, (5) low spatial resolution, and (6) similarity in intensity and texture amongst gas, bone, and connective tissues at several inter‐object interfaces. These issues make multi‐organ segmentation in dMRI images very challenging (Figure 1). To elucidate (6), the peripheral lungs can be difficult to separate from surrounding tissues, portions of the liver can be difficult to distinguish from the stomach and spleen, portions of the kidneys abut muscles subjacent to the spine, and portions of the spleen are adjacent to the stomach and left kidney.

**FIGURE 2:**
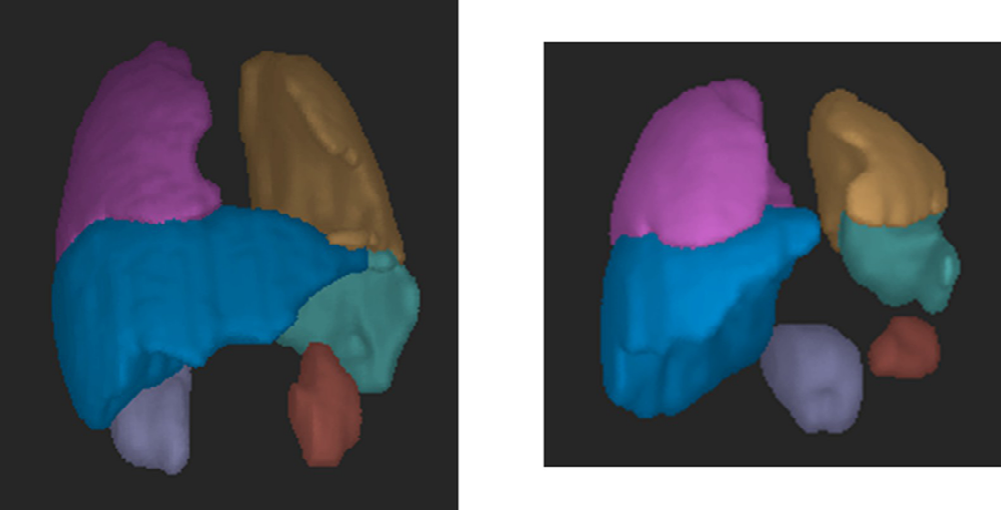
Three‐dimensional rendering of ground truth segmentation of left lung (yellow), right lung (pink), left kidney (red), right kidney (purple), liver (blue), and spleen (green) during end‐inspiration for (a) a 6.2‐year‐old female near‐normal subject and (b) 4.3‐year‐old female TIS patient.

Dynamic MRI acquisitions are inherently four‐dimensional, with the dimensions being space (in three dimensions) and time. In our dMRI acquisition, a sagittal slice MR image at a fixed location is first acquired continuously for a specified duration (typically over 10 respiratory cycles) while the subject is breathing naturally, and then the next sagittal slice is captured for the next specified duration, and so on until the right‐to‐left width of the entire thoraco‐abdominal region is fully covered. To segment the thoraco‐abdominal organs, we first perform a 4D construction of the body region image representing the dynamic body region over one respiratory cycle via an optical flux strategy,^17^ and then segment the 3D organs (Figure 2) in the 3D images corresponding to specified respiratory phases such as the end‐inspiration (EI) and end‐expiration (EE) phases. In this paper, we present a novel and unique system to address the problem of multi‐organ segmentation from dMRI acquisitions of the thoraco‐abdominal region. Based on the relationship between the images in the training and testing sets with respect to the respiratory phases of the images and the subjects to which the images belong, one major contribution of this paper lies in the different scenarios of training and testing that we extensively considered and evaluated for our experiments.

A preliminary version of this work was presented at the SPIE 2023 Medical Imaging Conference, whose proceedings contained a very abbreviated version of this work. The conference paper^18^ differs from the current paper in the following manner.

1. The 3D images at EI were utilized in reference.^18^ The current paper utilizes images at multiple (two (EI and EE) and greater than two) respiratory phases.
2. The conference paper focused on the large organs—the left lung and the right lung only. The current paper includes the more challenging left kidney, right kidney, liver, spleen, and thoraco‐abdominal skin outer boundary as well.
3. This paper includes further expansions. For example, we show how additional information from one respiratory phase can be considered for the delineation of the same object in the image of the same test subject in a different respiratory phase.
4. Our experimental evaluation involves a significant expansion over the conference paper, with a much larger data set (189 vs. 95 in the conference paper) and additional experiments involving repeated scans to show the consistency of performance of the proposed method.

## 2 RELATED WORK

The articles^5^, ^6^, ^7^, ^8^, ^9^, ^10^, ^11^, ^12^, ^13^, ^14^, ^19^ were found to be relevant to dMRI acquisitions for medical imaging applications. These can be grouped into four categories. (A) articles that review the viability of dMRI for specific clinical applications. (B) articles that discuss dMRI for the measurement of physiological parameters with the help of segmentation algorithms. (C) articles that discuss dMRI in the context of clinical applications, but with manual analysis of dMRI images. (D) Other articles, such as those discussing image reconstruction in dMRI. We give a brief overview of the articles in these categories.

Articles^5^, ^6^ in Category A are not related to the segmentation of objects in dMRI images. Specifically, the authors in^5^ tried to show how dynamic contrast‐enhanced MRI (DCE‐MRI) is an attractive imaging modality for measuring peripheral perfusion, other diverse microvascular parameters such as vessel permeability and fluid volume fractions, and tissue perfusion. The authors in^6^ discussed techniques of using dMRI for evaluating joint motion.

The articles^7^, ^8^, ^9^, ^10^, ^11^ in category B deal with the segmentation of a single object of interest in dMRI images. Articles^8^, ^9^, ^10^, ^11^ did not elucidate details of their segmentation methods. The authors in^7^ used dMRI to assess pelvic organ prolapse with segmentation of the vertebral shape. Since we are dealing with volumetric objects compared to linear objects, we cannot directly apply their method to segment the organs in **O**. The authors in^8^ demonstrated the feasibility of quantitative cerebral blood flow (CBF) measurements during supine bicycling exercise with pseudo‐continuous arterial spin labeling MRI acquired at 3T. The authors in^9^ utilized classical segmentation algorithms to segment foci of tumors in the prostate gland in the dMRI image. The authors in^10^ tried to indirectly measure local changes in CBF, blood volume, and blood oxygenation from neuronal activity via segmentation in the dMRI image. Lastly, the authors in^11^ proposed a method to segment and remove the skin from the dMRI image of the breast to improve the clarity of breast tissue in the dMRI image for further diagnosis. We reiterate that the articles cited in this paragraph deal with the segmentation of a single object of interest in dMRI images and do not deal with dynamic or moving thoraco‐abdominal organs, noting that some of the dMRI images pertain to studying the kinetics of administered contrast material rather than the dynamic motion of organs.

The articles^12^, ^13^, ^14^ use manual segmentations of relevant objects, such as the chest wall, lungs, and diaphragm in dMRI images, to measure parameters of interest in normal adult subjects. Note that the pediatric dMRI we deal with is, in general, more challenging during acquisition and subsequent analysis due to a higher respiratory rate, worse image quality, and difficulty in subject compliance. A method developed for adult images cannot be assumed to be generalizable to those obtained in pediatric subjects.

We found one article^19^ that belongs to category D. The authors in^19^ discuss a novel approach for reconstructing the dMRI image quickly from *k*‐space and spatial prior knowledge via a multi‐supervised network, which they call “DIMENSION”. However, article^19^ does not deal with segmentation.

The authors of^15^ proposed a deep neural network for segmentation of the lungs from dMRI sagittal acquisitions of (near‐) normal pediatric subjects using a U‐Net architecture. The article^16^ uses atlas‐based segmentation approaches for the lungs in dMRI images with a worse spatial resolution (2.81 × 2.81 × 4 mm) compared to our dMRI images (∼1 × 1 × 6 mm). It is not disclosed whether the subjects in^16^ are adults or children. In the current paper, our segmentation approach is a two‐step approach: recognition and delineation. The recognition step localizes the object of interest with bounding boxes, and the delineation step marks the outline of the object of interest in the bounding box. The delineation step uses the neural networks in,^20^, ^21^ where the encoder‐decoder architecture is enhanced with different modules such as the Path Aggregation Network (PAN)^22^ and the Dual Attention Network (DAN).^23^ We adopted this enhanced architecture as we are also dealing with the segmentation of the left kidney, right kidney, liver, and spleen, which are more challenging (given poor contrast and inconsistent intensity meanings) to handle than the segmentation of the lungs.

We also checked comparable deep learning methodologies in the literature by referring to survey papers.^24^, ^25^, ^26^, ^27^, ^28^ We noticed that Mask R‐CNN^29^ is a conceptually similar approach to segmentation using localization followed by mask prediction, although there are two important differences between Mask R‐CNN and our approach to segmentation. First, the training of localization and mask prediction modules in Mask R‐CNN is coupled (dependent on one another), whereas in our approach, the training of recognition and delineation networks is decoupled. Second, the mask prediction module in Mask R‐CNN uses a simple fully convolutional framework, whereas we use a dedicated encoder‐decoder architecture for the delineation step in our approach. This splitting of the heavy‐duty task of segmentation into decoupled modules of recognition and delineation is necessary to handle the challenging dMRI images because of the aforementioned problems (refer Section 1).

For the sake of completeness, we also refer to nn U‐Net,^30^ which uses a cascade of two 3D U‐Nets and which has achieved better performance than other network architectures in the Brain Tumor Segmentation Challenge Dataset.^31^ The nn U‐Net tunes parameters related to cropping, re‐sampling, normalization, data augmentation, and patch sampling based on the object of interest to be segmented in medical imaging modalities such as computed tomography (CT) and MRI. However, this network does not have a prior component for localizing the object and uses the 3D U‐Net architecture directly for segmenting the object within patches.

From the above discussion, we conclude that, except for,^15^, ^16^ the problem of multi‐organ (greater than 2 organs) segmentation in the thoraco‐abdominal region of dMRI acquisitions, pediatric or adult, has not been addressed before.

## 3 METHODS

**FIGURE 3:**
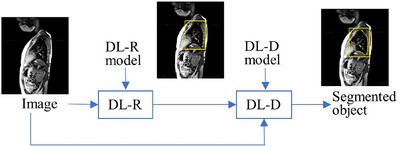
Illustration of our segmentation pipeline. The deep learning recognition (DL‐R) module is used for the recognition step, and the deep learning delineation (DL‐D) module is utilized for the delineation step.

We perform segmentation in two steps: a recognition step and a delineation step (Figure 3). In recognition, we try to obtain a rough idea of the location of the object of interest in the unseen image with the help of bounding boxes. In delineation, the approach marks the outline of the object of interest within the bounding box. We have utilized deep learning recognition (DL‐R) and deep learning delineation (DL‐D) networks for recognition and delineation. We give a description of DL‐R and DL‐D in Sections 3.1 and 3.2, respectively. In the next subsection, we discuss details of the dataset that was used for all experiments presented in this paper.

### 3.1 Data acquisition and pre‐processing

#### 3.1.1 dMRI scans

The dMRI scan data were acquired from 189, six to 20‐year‐olds, healthy children under an ongoing prospective research study protocol approved by the Institutional Review Board at the Children's Hospital of Philadelphia (CHOP) and University of Pennsylvania, along with a Health Insurance Portability and Accountability Act waiver. We excluded scans with significant body movement during scanning or with obvious image artifacts. The thoraco‐abdominal dMRI protocol includes a 3T MRI scanner (Verio, Siemens, Erlangen, Germany) using a balanced steady‐state gradient recalled echo (True‐FISP) sequence with acquisition and reconstruction parameters of TR = 3.82 ms, TE = 1.91 ms, flip angle 76 degrees, bandwidth 258 Hz, 320 × 320 matrix, and voxel size ∼1 × 1 × 6 mm^3^. For each sagittal location across the thorax, 80 image slices were obtained over several tidal breathing cycles at ∼480 ms/slice. On average, 35 sagittal locations across the chest and abdomen were imaged. Therefore, a total of 2800 (35 × 80) 2D MRI slices were acquired per subject.

#### 3.1.2 4D construction

Given the dMRI scan for each subject, a small set of 175–320 slices representing one 4D volume over one respiratory cycle is selected from the 2800 2D free‐breathing dMRI image slices using an optical flux‐based optimization method to represent the dynamic thorax and abdomen of the subject.^17^

#### 3.1.3 Image intensity standardization

MRI signal intensities in the 4D constructed image are standardized^32^ to a standard intensity scale to facilitate object segmentation and analysis. Intensity standardization enables voxel intensity values to have a similar numeric meaning for each type of tissue within the same subject, across subjects, in repeat scans on the same scanner, and across different scanners.^32^, ^33^

#### 3.1.4 Creating ground truth segmentations

Following the principles outlined in,^34^ we created clinically meaningful and computationally feasible definitions of the thoraco‐abdominal body region and objects considered in this application to make the models anatomically specific and to minimize inter‐tracer variability while creating the ground truth (GT) masks of the objects. We define the thoraco‐abdominal body region considered in this application as extending from 15 mm superior to the lung apices to the inferior aspect of the kidneys. Similarly, each object was defined in terms of which substructures are to be included/excluded. A board‐certified radiologist with more than 25 years of experience (D.A.T.) trained students, post‐doctoral fellows, engineers, and medical interns (R.K., L.T., S.M., Y.A., C.W., M.A., M.H.) in the anatomic and dMRI radiological appearance of the relevant objects of interest. Following training, the 7 organs of our focus in the 189 dMRI acquisitions were all segmented manually by the above individuals through the use of the open‐source software CAVASS^35^ in the EE and EI time points of the respiratory cycle. This yielded a total of 2646 (=189 × 7 × 2) 3D object samples for our cohort.

### 3.2 Segmentation: DL‐R^20^

**FIGURE 4:**
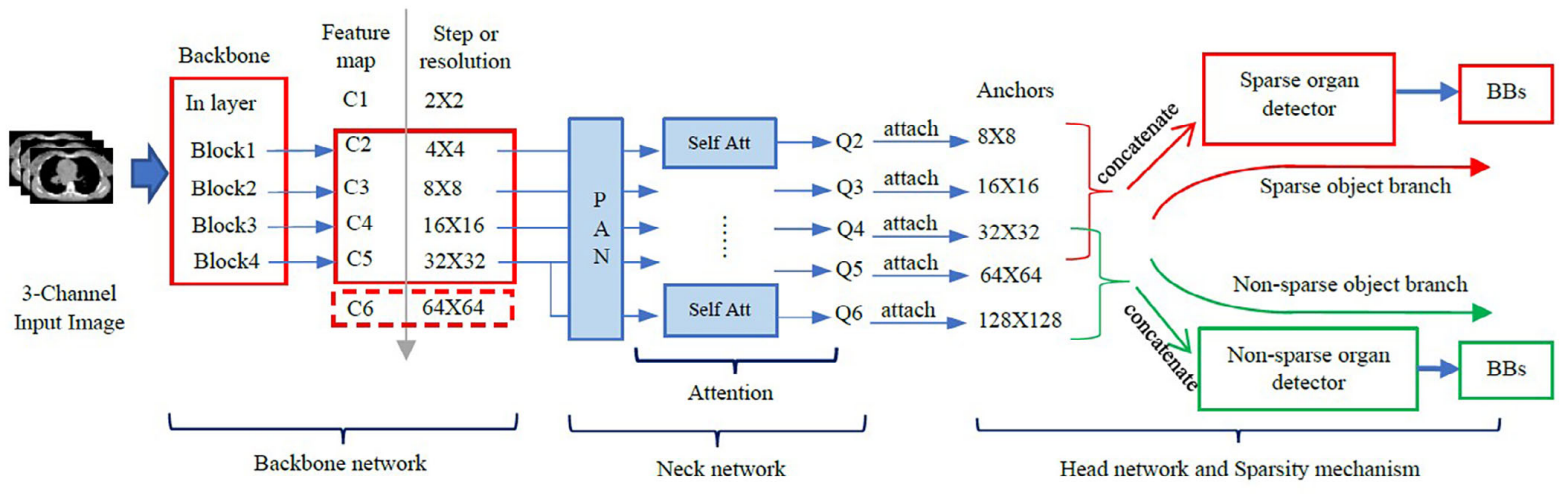
Overall architecture of DL‐R.^20^ Att = attention; BBs = bounding boxes; PAN = path aggregation network.

The DL‐R module (Figure 4) consists of a backbone network, neck network, and head network. The backbone network uses a 3‐channel version of a gray‐scale dMRI slice as input and pre‐trained model weights based on ResNet^36^ and DenseNet,^37^ yielding four feature maps (C2, C3, C4, and C5), which are input to the neck network. The maps C2 and C5 capture lower‐level and higher‐level textural information, respectively, compared to C3 and C4.

The neck network is based on PAN^22^ and DAN^23^ architectures. The PAN architecture creates maps referred to by Q4, Q5, and Q6 by merging C2, C3, C4, and C5 using bottom‐up connections, top‐down connections, and lateral connections. The DAN is used to create prediction maps, which contain the information dependency across the spatial and channel dimensions of the maps Q4, Q5, and Q6. The maps Q4, Q5, Q6, and the prediction maps are taken as input to the head network.

The head network recognizes the non‐sparse organs with the maps Q4, Q5, and Q6 by associating them with anchor sizes 32 × 32, 64 × 64, and 128 × 128, respectively. This recognition is further refined by utilizing the prediction maps and the anchors with the help of convolutional layers. The output of DL‐R is a bounding box (BB) in those sagittal slices that are identified to contain the objects of interest.

Useful features for recognition that are conspicuous at a large scale, such as the presence of bifurcations of blood vessels in the liver or the appearance of the chambers of the heart in the dMRI image, are handled by C5, while similar features, if present inconspicuously at a local scale, are handled by C2. This makes the DL‐R approach appealing as it integrates different types of information at varying scales in its design.

For training and testing the DL‐R model, intensity standardization (IS)^33^ of the images is performed first. The DL‐R model takes three thresholds as input for transforming a sagittal slice to the 3 (color)‐channel 2D image (please refer to the second paragraph of this subsection). The motivation behind creating this 3‐channel image is to roughly visually depict different compositions of the body region based on intensity values alone. We have chosen the first color channel to represent low intensity objects (such as gas and cortical bone), the second color channel to represent medium intensity objects (such as soft tissues of the skeletal muscles and visceral organs), and the third color channel to represent high intensity objects (such as adipose tissue, cardiac chambers, and blood vessels). Note that for this process to be meaningful, IS should be performed first; otherwise, the object classes may be mixed up.

**FIGURE 5:**
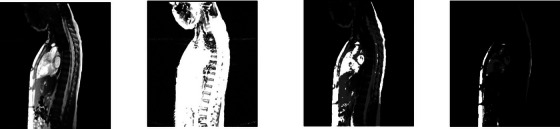
Color channel management in dMRI images. (a) Original representative sagittal image of a thoraco‐abdominal dMRI scan acquisition. (b) The first channel represents low‐intensity objects. (c) The second channel represents medium‐intensity objects. (d) The third channel represents high‐intensity objects.

For each color channel (Figure 5), two thresholds (an upper threshold and a lower threshold) are chosen to roughly contain the intensities of interest. Based on our visual inspection of the histograms of the pixel intensity values of the objects in **O** in the IS images, we have chosen the three intensity thresholds as 150, 750, and 1500 (on a standardized intensity scale of 0–4095) For example, consider a channel that uses a lower threshold (L) and an upper threshold (U). If the pixel value in the IS image is *y*, then we transform *y* to 0 if it is less than L. If *y* lies between L and U, then it is transformed to 255*(*y*‐L)/(U‐L). If *y* is greater than U, then it is transformed to 255. [L, U] for the three channels are: [0, 150), [150, 750), and [750, 1500], respectively.

The DL‐R module is optimized using an Adam optimizer with a learning rate of 0.00001. The Focal Loss^38^ function is utilized for optimization of the DL‐R module.

### 3.3 DL‐D^21^

**FIGURE 6:**
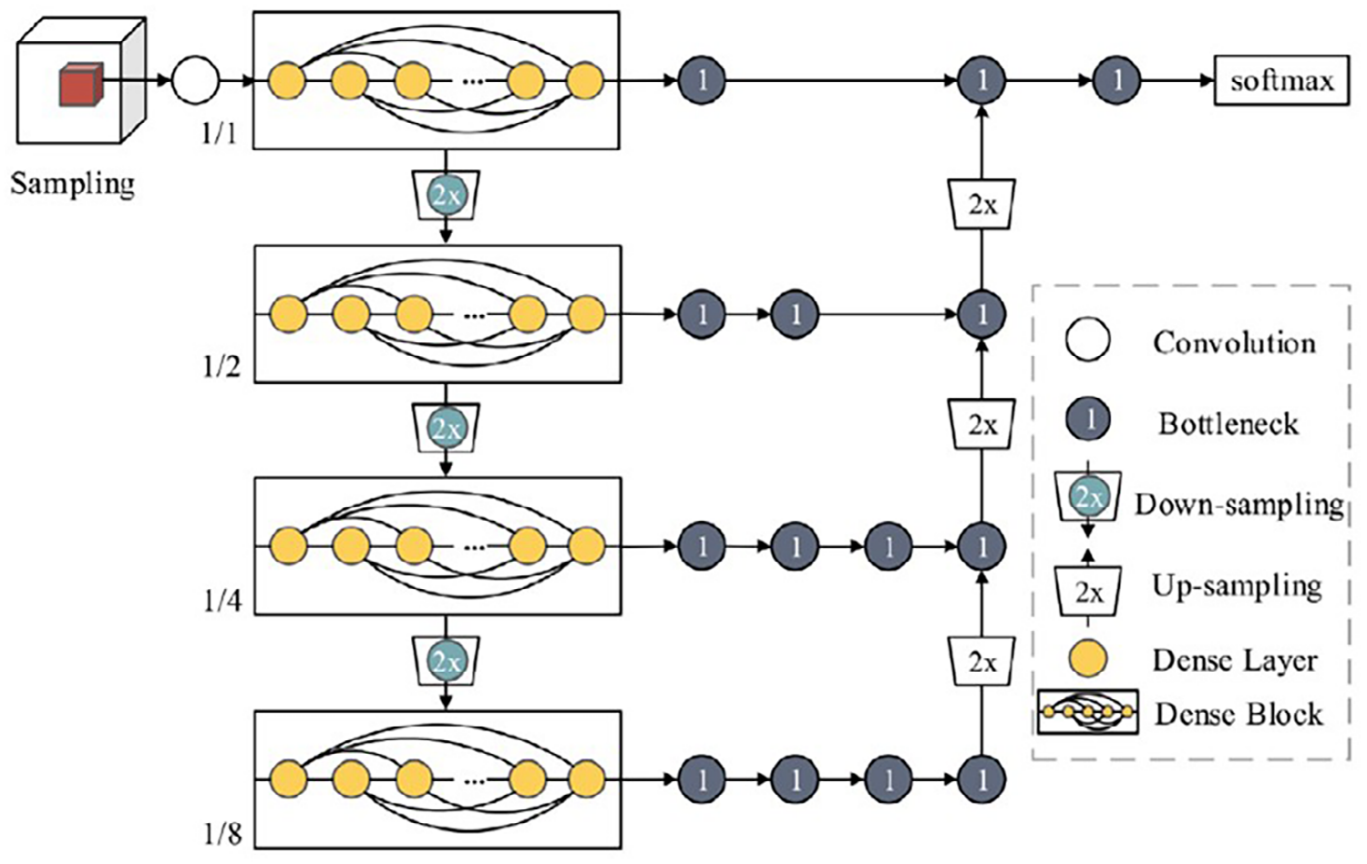
Architecture of ABCNet.^21^

This module utilizes a network called ABCNet,^21^ which was originally designed to delineate the different types of body tissues: subcutaneous adipose tissue, visceral adipose tissue, skeletal muscle tissue, and skeletal tissue from low‐dose axial CT images of the body torso. The design of ABCNet is similar to an encoder‐decoder architecture (Figure 6).

The fundamental unit of ABCNet is referred to as BasicConv, which comprises concatenation, batch normalization, activation, and convolution in succession. Bottleneck is a special case of BasicConv with a convolutional kernel of 1 × 1 × 1. There are four DenseBlocks^37^ used in the encoder‐decoder architecture of ABCNet. The deeper the DenseBlock, the more high‐level information it extracts from the input image. Each DenseBlock of ABCNet is composed of Dense Layers, which are themselves composed of Bottleneck and BasicConv with a kernel size of 3 × 3 × 3 in succession. The Bottleneck, because of its lower convolutional kernel size, keeps the number of parameters less and simultaneously acts as a feature extractor through the normalization and activation functions of its BasicConv architecture.

The ABCNet model uses a Dice coefficient‐based loss function for training its model and selects patches randomly from within and slightly around the ground truth in the images of the seen dataset during training. During testing, the patches are selected from within and slightly around the BB (from the recognition step) in the images of the unseen dataset. The output of ABCNet is the prediction map of the object from the decoder. This prediction map is binarized using a threshold to yield the final segmentation of the object. Unlike existing encoder‐decoder architectures (DeepMedic,^39^ Dense V‐Net,^40^ V‐Net,^41^ and 3D U‐Net^42^), which have typically 12 to 31 layers and 1 million to 80 million parameters, ABCNet has 118 layers with only 1.4 million parameters. The usage of ABCNet is thus attractive because of its deeper architecture with a lower number of parameters.

We used intensity normalization on the **IS** images before using them for training and testing the DL‐D module. For intensity normalization, the *Z*‐score method was utilized. The *Z*‐score utilizes the mean (and standard deviation) of the standardized pixel values belonging to the object over all images in the training set. Let this mean and standard deviation be denoted by *μ* and *σ*, respectively. A pixel value *x* in an image of the training set or in the testing set is transformed to a new value y by the relation *y* = (*x*‐*μ*)/*σ*.

The patch size, which is an input to the DL‐D module, is chosen as 72 × 72 × 24 voxels for large organs such as the left lung, right lung, liver, and thoraco‐abdominal skin outer boundary, and as 72 × 72 × 16 voxels for smaller organs such as the spleen, left kidney, and right kidney. Smaller patches lead to a reduction in the delineation accuracy of DL‐D. Larger patches require a large amount of memory, sometimes exceeding the workstation's memory capacity and exponentially increasing the time required for training the DL‐R and DL‐D models.

The DL‐D module is trained for 50 epochs with 200 steps per epoch. A batch size of four is utilized for a mini‐batch gradient descent for optimization. The initial learning rate is set to 0.01, which is reduced further to 0.00001 by the cosine annealing strategy.

### 3.4 Scenarios in segmentation

If we can create GT or close to GT segmentations (requiring minimal post hoc corrections) through our pipeline, then this would be very useful, given that the manual creation of GT segmentations from scratch for all time points (phases) in dMRI images is labor‐intensive and impractical. We evidently need to train our networks for obtaining the DL‐R and DL‐D models to be utilized for creating the GT. Certain scenarios during these training and testing processes would arise as discussed below.

We can use two parameters to describe the images in the training set or testing set. The first parameter is the subject (PSub) to which an image belongs. The second parameter (PResp) is the respiratory phase to which the image belongs. We consider only those scenarios for experimentation such that no image in the training set and no image in the testing set has both PSub and PResp identical.

The problem of segmenting an object at any temporal phase (Q) for a subject *x* given the GT segmentation for one phase (P ≠ Q) for *x*, is also legitimate and practically very relevant. We conducted experiments where we assume that we have the GT segmentation for an organ of interest in an image of the test subject at a respiratory phase P that is different from the respiratory phase Q, in which we are trying to segment the organ. In these experiments, initially, the BBs for object delineation at a respiratory phase P1 closest to P come from enlarged tight‐fitting BBs around the GT segmentations at P. The BBs for the delineation at the next respiratory phase, P2, closest to P1 come from enlarged, tight‐fitting BBs around the GT segmentations at P1. This process continues iteratively until we have the delineations for the organs in the image of the test subject at respiratory phase Q. The results of such experiments are shown in the next section.

## 4 EXPERIMENTS AND RESULTS

As summarized in Table 1, we have conducted 10 experiments (Exp. 0–Exp. 9) to analyze the behavior of the whole pipeline. In particular, we focused on the 3D images corresponding to EE and EI respiratory phases since they are critical for analyzing lung tidal volumes. We have evaluated the DL‐R approach based on the location error (*LE*), which is defined as the distance between the centroids of the BB from the recognition step and the tight‐fitting BB around the GT true object. The performance of the DL‐D approach was evaluated based on the Dice coefficient (*DC*) and the mean‐Hausdorff (mean‐*HD*) distance. The *DC* is calculated as 2n(X∩Y)/(n(X)+n(Y)), where n(X) represents the number of elements in *X*. The notations *X* and *Y* represent the set of pixels that belong to the GT and the auto‐segmentation marking, respectively, of the object. The mean‐*HD* between two sets of points *M* and *N* is defined as the average of d_avg_(M, N) and d_avg_(N, M), where d_avg_(M, N) is the average of the distances between points in *M* to their nearest neighbor in *N*. The *LE* and the mean‐*HD* are expressed in units of mm, while *DC* is dimensionless.

**TABLE 1:** Summary of related information about the 9 experiments presented in this paper. EI = end‐inspiration; EE = end‐expiration.

Experiment	Approach	During testing, do we use additional information about a test image of a subject such as ground truth of an image of the subject at a respiratory phase which is different from the phase of the test image?	Number of images in training set	Number of images in validation set	Number of images in testing set	Respiratory phase of images in training and validation sets	Respiratory phase of images in testing set	Are subjects in the testing set identical to those in training and validation sets?	Are respiratory phases of images in training and validation sets identical to those of testing set?	Are all organs (lungs, kidneys, liver, spleen, and thoraco‐abdominal skin outer boundary) segmented?
Exp. 0	Proposed	No	72	10	13	EI	EI	No	Yes	Yes
Exp. 1			89	10	90		EI		Yes	
Exp. 2					90		EE		No	
Exp. 3					99		EE	Yes	No	
Exp. 4		Yes			90		EE	No	No	
Exp. 5					90		EI		Yes	
Exp. 6					99		EE	Yes	No	
Exp. 7		No			20		EI and EE	No	Mixed	No (only left lung and right lung)
Exp. 8	[15]		36 images for training and validation	140	EE	EI (70) and EE (70)	No	Mixed		No (only lungs)
Exp. 9	[29]	No	99	90	EI	EI	No	Yes		No (only lungs, kidneys, liver, and spleen)

### 4.1 Exp. 0: Experiment which utilizes (3D) images at a single respiratory phase (EI) with data augmentation

In this experiment, we explored different data augmentation techniques using reflection of the dMRI images across a 2D plane [axial (transverse), coronal, or sagittal] or reflection across a particular combination of the 2D planes. We examine each organ of interest to see what reflection mode makes anatomic sense for that organ, and examine the delineation output by DL‐D for all methods of data augmentation. In Table 2, “0”, “1”, and “2” mean reflection is made across the axial (transverse), coronal, or sagittal plane, respectively, and a serial listing of these numbers indicates a series of reflections (e.g., “01” means that reflection is first made across the axial (transverse) plane, and then across the coronal plane). The last column of Table 2 indicates no reflection.

**TABLE 2:** Mean (1st value) and standard deviation (SD, 2nd value) of DC over the testing data set are listed for the 7 organs for the different data augmentation strategies. “0”, “1”, “2” mean reflection about the axial (transverse), coronal, and sagittal planes, respectively. Multiple digits indicate sequential reflections involving multiple planes. For example, “01” means reflection is first made across the axial (transverse) plane and then across the coronal plane.

Organ	“0”	“1”	“2”	“01”	“12”	“02”	“012”	None
Left lung	0.947 ± 0.021	0.944 ± 0.013	0.911 ± 0.085	0.947 ± 0.024	0.938 ± 0.033	0.942 ± 0.028	0.905 ± 0.052	**0.948** ** ± 0.021**
Right lung	0.945 ± 0.019	0.956 ± 0.013	0.954 ± 0.024	0.907 ± 0.047	0.933 ± 0.032	0.946 ± 0.021	0.942 ± 0.032	**0.958** ** ± 0.008**
Left kidney	0.805 ± 0.092	0.772 ± 0.142	0.799 ± 0.095	0.785 ± 0.111	0.840 ± 0.029	**0.855** ** ± 0.029**	0.774 ± 0.210	0.820 ± 0.071
Right kidney	**0.871** ** ± 0.048**	0.806 ± 0.126	0.793 ± 0.238	0.685 ± 0.314	0.780 ± 0.192	0.781 ± 0.239	0.839 ± 0.061	0.855 ± 0.089
Liver	0.865 ± 0.052	0.856 ± 0.044	0.823 ± 0.056	0.866 ± 0.070	0.895 ± 0.023	**0.911** ** ± 0.019**	0.893 ± 0.029	0.891 ± 0.026
Spleen	0.792 ± 0.109	**0.824** ** ± 0.053**	0.818 ± 0.069	0.802 ± 0.077	0.783 ± 0.102	0.811 ± 0.066	0.803 ± 0.067	0.791 ± 0.089
Thoraco‐abdominal skin outer boundary	**0.911** ** ± 0.025**	0.896 ± 0.032	0.906 ± 0.031	0.893 ± 0.020	0.888 ± 0.032	0.902 ± 0.032	0.887 ± 0.033	0.908 ± 0.027

dMRI images of 72, 10, and 13 subjects at the EI respiratory phase were used for training, validation, and testing, respectively, of the DL‐D module. In Table 2, the average *DC* indicates that for each of the seven organs, a particular method of reflection is optimal. However, such data augmentation techniques are not meaningful in the context of our thoraco‐abdominal organ segmentation problem, as the meaning behind the appearance of the organs in the reflected image changes with respect to the meaning of the appearance of the organs in the original image. The results of the last column of Table 2 with no reflection show excellent *DC* for most of the organs, suggesting that this augmentation method is not useful.

### 4.2 Exp. 1–3: Experiments which utilize (3D) images at two respiratory phases (EI and EE)

Based on the first paragraph of section 3.3, we explore three scenarios denoted F, G, and H (see Table 3). In scenario F, PResp is identical between the training and testing sets, but PSub is different. In G, PResp and PSub are different between the training and testing sets. In H, PResp is different, but PSub is identical between the training and testing sets. We utilize 89 images at EI for training and 10 images at EI for validation in F, G, and H. For testing, we utilize in F: the images of 90 (=189‐89‐10) different (remaining) subjects at EI, in G: the images of the aforementioned 90 subjects at EE, and in H: the images of the aforesaid 99 (=89+10) subjects but at EE.

**TABLE 3:** Partitioning of 189 subjects for the evaluation of DL‐R and DL‐D modules for Exp. 1–3.

Number of subjects	Respiratory phase of image	Purpose	Scenario	PResp	PSub
99	EI	Training (89) and Validation (10)	F, G, and H	—	—
	EE	Testing	H	Different	Same
90	EI		F	Same	Different
	EE		G	Different	Different

In Exp. 1–3, we utilize scenarios F–H, respectively, where the results are summarized in Table 4 for DL‐R and DL‐D, including *DC* and mean‐*HD*. The recognition error for DL‐R is expressed in terms of location error *LE*. Note that in Exp. 3, an image in the training set and an image in the testing set can belong to the same subject dMRI acquisition. However, these two images belong to different respiratory phases (as EE for the testing set and as EI for the training set).

**TABLE 4:** Recognition location error (LE, mean ± SD) in mm of DL‐R and delineation results (Dice coefficient (DC), mean ± SD) and (mean‐Hausdorff distance (mean‐HD), mean ± SD) in mm of DL‐D on images at end inspiration (EI) and end expiration (EE) for experiments Exp. 1–3. The least LE, greatest DC, and least mean‐HD under each column are shown in bold. For further details, please refer subsection 4.2.

Test	Measure	Left lung	Right lung	Left kidney	Right kidney	Liver	Spleen	Thoraco‐abdominal skin outer boundary
Exp. 1 (F: 90 EI)	*LE*	5.69 ± 3.23	6.31 ± 5.81	4.66 ± 2.50	5.16 ± 8.12	7.69 ± 6.09	5.50 ± 3.51	7.18 ± 4.22
	*DC*	0.936 ± 0.018	0.930 ± 0.039	0.808 ± 0.094	0.828 ± 0.072	0.896 ± 0.053	0.807 ± 0.075	0.929 ± 0.029
	Mean‐*HD*	**0.52** ** ± 0.24**	1.10 ± 2.57	**1.50** ** ± 1.61**	1.43 ± 1.57	2.50 ± 3.84	**1.15** ** ± 1.12**	3.79 ± 2.31
Exp. 2 (G: 90 EE)	*LE*	5.35 ± 3.38	5.20 ± 2.95	4.15 ± 2.45	4.41 ± 2.80	10.21 ± 8.27	5.83 ± 3.52	7.11 ± 4.25
	*DC*	0.924 ± 0.033	0.923 ± 0.041	0.811 ± 0.069	0.833 ± 0.082	0.899 ± 0.053	0.826 ± 0.050	0.925 ± 0.028
	Mean‐*HD*	0.65 ± 0.63	0.83 ± 1.00	1.85 ± 2.25	2.12 ± 4.17	**2.34** ** ± 2.96**	1.38 ± 0.86	**2.14** ** ± 1.09**
Exp. 3 (H: 99 EE)	*LE*	**4.73** ** ± 3.44**	**4.90** ** ± 2.63**	**3.79** ** ± 2.16**	**3.70** ** ± 1.93**	**6.94** ** ± 5.76**	**4.31** ** ± 2.17**	**4.09** ** ± 2.85**
	*DC*	**0.943** ** ± 0.026**	**0.939** ** ± 0.028**	**0.833** ** ± 0.056**	**0.843** ** ± 0.077**	**0.909** ** ± 0.023**	**0.830** ** ± 0.057**	**0.937** ** ± 0.024**
	Mean‐*HD*	0.67 ± 0.96	**0.59** ** ± 0.37**	1.98 ± 2.65	**1.29** ** ± 1.85**	2.64 ± 5.41	3.02 ± 8.73	3.14 ± 1.69

We notice from the results in Table 4 that out of the three experiments, Exp. 3 has the least average *LE* for all 7 organs. This observation aligns with our intuition that if we have additional information about the test image (such as the GT in the image of a test subject at a particular respiratory phase), and use it in training DL‐R, we will obtain better recognition results in the image of the same subject at a different respiratory phase.

Among Exp. 1, Exp. 2, and Exp. 3, we notice from the results in Table 4 that Exp. 3 fares the best for all organs with respect to *DC*, and for the right lung and right kidney with respect to mean‐*HD*, whereas Exp. 1 fares the best for the left lung, left kidney, and spleen, and Exp. 2 fares the best for the liver and thoraco‐abdominal skin outer boundary with respect to mean‐*HD*. The competitive performance of Exp. 3 is in accordance with our intuition that if an image (say *A*) of a subject at a particular respiratory phase is included with its associated GT segmentation in the training of DL‐D, then the delineation performance of DL‐D for an image of the same subject at a different respiratory phase will be better compared to its performance when *A* is not used in training.

### 4.3 Exp. 4–6: Experiments which utilize (3D) images at multiple (> 2) respiratory phases (EI, EE, and intermediate phases)

Based on the third paragraph of section 3.3, we present the performance of DL‐D for the delineation of the 7 organs in images of the 90 subjects at EE in an incremental manner (i.e., Exp. 4 where Q = EE and P = EI). Of these 90 subjects, 29 had one intermediate respiratory phase between EI and EE, 57 had 2 intermediate respiratory phases between EI and EE, and 4 had 3 intermediate respiratory phases between EI and EE. The number of intermediate respiratory phases is variable because of the different respiratory rates of the subjects,^17^ which affects the quality of the image, and which in turn determines how many respiratory phases within a respiratory cycle can be utilized to reliably reconstruct the image of the body region. The training (and validation) set consisted of images of the 89 (and 10) subjects at EI. The results in terms of mean (and SD) of Dice coefficients over all 90 test subjects for 7 organs are shown in the second row (Exp. 4) of Table 5. The corresponding mean (and SD) of the mean‐*HD* values are shown in Table S1.

**TABLE 5:** Performance (DC, mean ± SD) of DL‐D for delineation of left lung, right lung, left kidney, right kidney, liver, spleen, and thoraco‐abdominal skin outer boundary in an incremental manner in three different experiments. The highest DC under a column is shown in bold.

Experiment	Left lung	Right lung	Left kidney	Right kidney	Liver	Spleen	Thoraco‐abdominal skin outer boundary
Exp. 4 (90 EI to EE)	0.937 ± 0.018	**0.929** ** ± 0.040**	**0.820** ** ± 0.077**	0.831 ± 0.086	0.898 ± 0.048	0.808 ± 0.073	**0.949** ** ± 0.028**
Exp. 5 (90 EE to EI)	0.922 ± 0.035	0.923 ± 0.039	0.814 ± 0.066	0.830 ± 0.087	0.897 ± 0.039	0.811 ± 0.060	0.936 ± 0.027
Exp. 6 (99 EI to EE)	**0.942** ** ± 0.026**	0.921 ± 0.052	0.816 ± 0.070	**0.841** ** ± 0.097**	**0.907** ** ± 0.020**	**0.813** ** ± 0.071**	0.945 ± 0.026

In Exp. 5, we test the delineation of the 7 organs on images of the 90 subjects at EI (Q = EI), using the GT segmentations of organs in the images of the aforesaid 90 subjects at EE (P = EE), in an incremental manner. The results are shown in the third row as Exp. 5 of Table 5 and in Table S2.

In Exp. 6, we test the DL‐D for the 7 organs on images of 99 subjects at EE (Q = EE) using the GT segmentations of the organs in the images of the aforesaid 99 subjects at EI (P = EI), in an incremental manner. Out of these 99 subjects, nine did not have images at intermediate respiratory phases. Amongst the remaining 90 (=99‐9) subjects, 28, 54, and eight had images at 1, 2, and 3 intermediate respiratory phases, respectively. The results are shown in (the fourth row as Exp. 6 of) Table 5 and Table S3.

Exp. 5 and Exp. 1 are similar in the sense that both try to delineate organs in images of 90 subjects at EI, and the same holds true for Exp. 4 and Exp. 2 at EE. The results of Exp. 5 in Table 5 are better by about 1% for the left kidney, right kidney, spleen, and thoraco‐abdominal skin outer boundary compared to those of Exp. 1 in Table 4. Except for the right kidney, liver, and spleen, Exp. 4 yielded better results than Exp. 2 by about 1% to 3%. Exp. 6 and Exp. 3 are similar in the sense that both try to delineate organs in images of 99 subjects at EE. Exp. 6 yields inferior results by about 2% for the right lung, left kidney, and spleen compared to those of Exp. 3. For the remaining organs, Exp. 6 and Exp. 3 perform in a statistically similar fashion.

We notice that despite using additional information about the test image in Table 5, the results did not significantly improve compared to those in Table 4 with respect to *DC*. We speculate that this is because the delineations at an intermediate respiratory phase are not sufficiently accurate to provide a correct BB for the delineation of the organs at the subsequent respiratory phase. We can expect this drawback to be minimal if the adjacent respiratory phases are closer to one another in phase. For example, if we look at the results in Tables S1–S3, we notice that the mean‐*HD* assumes the least value mostly at the “3” number of intermediate respiratory phases. This suggests that if we have images at a larger number of intermediate respiratory phases between EE and EI, then the incremental manner of testing DL‐D would demonstrate better performance. Experiments 7–9 are discussed in the Appendix.

**FIGURE 7:**
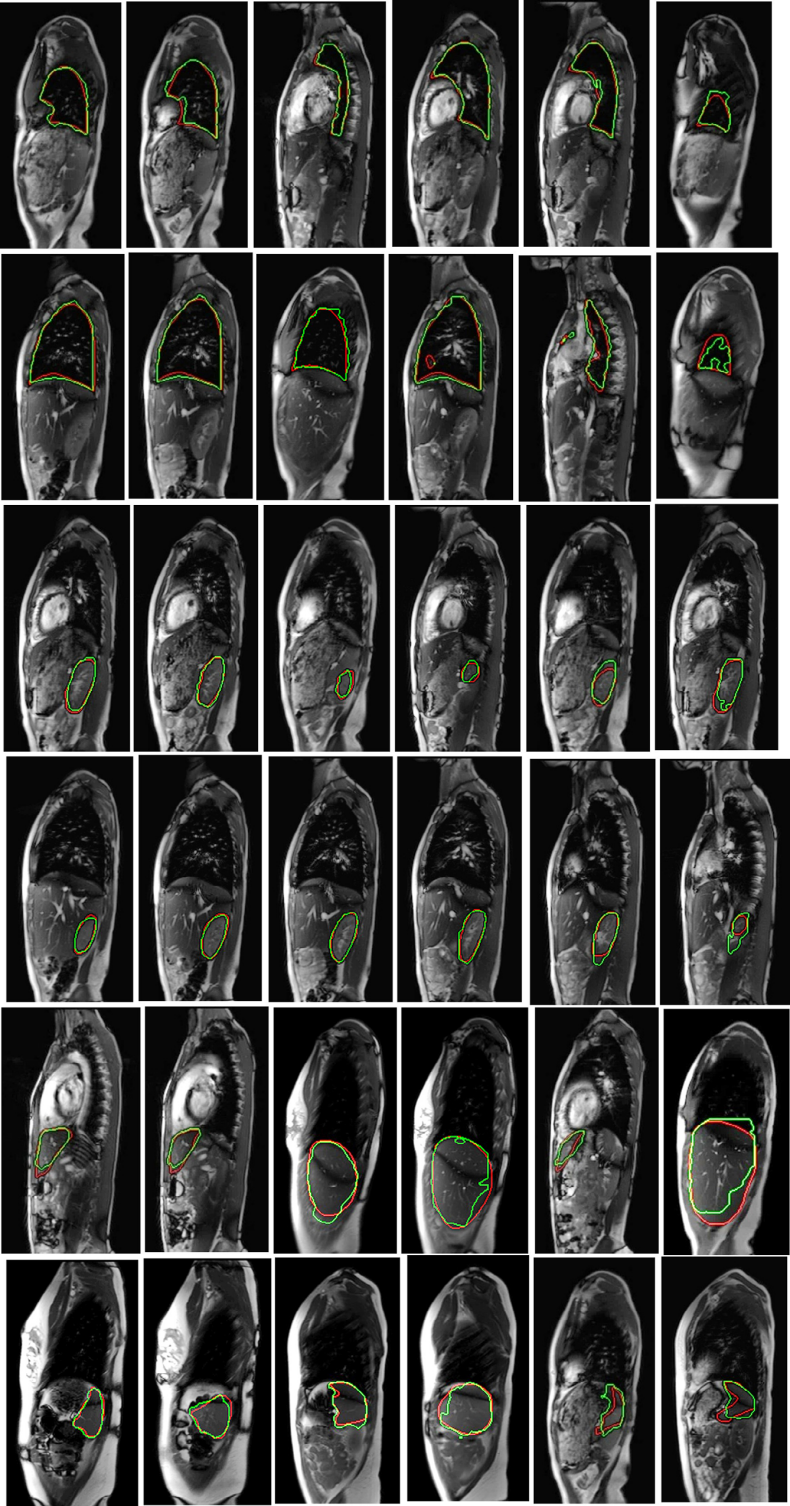
Each row shows the delineations for an organ (top to bottom: left lung, right lung, left kidney, right kidney, liver, and spleen) by the proposed auto‐segmentation algorithm (green) and by the expert human tracer (red) in six sagittal dMRI slices. The last column shows those cases where the delineations by the auto‐segmentation algorithm are relatively more off compared to the ground truth segmentations.

**FIGURE 8:**
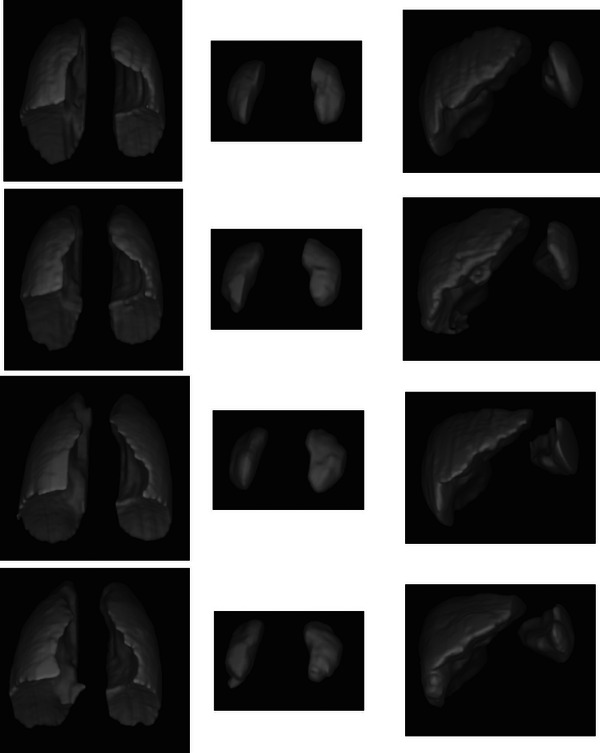
3D rendering of ground truth segmentations (1st row) and corresponding predictions (2nd row) of a subject (Subject 1) for left lung and right lung (1st column), left kidney and right kidney (2nd column), and liver and spleen (3rd column). Renderings obtained in a similar fashion are displayed for a second subject (Subject 2) in the 3rd row (ground truth) and 4th row (predictions).

**FIGURE 9:**
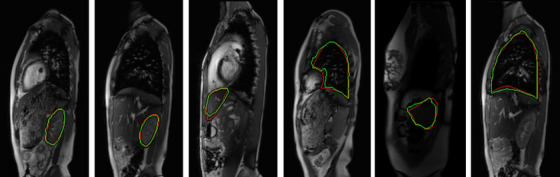
Images in the second column of Figure 7 have been displayed with their rendering based on the original pixel intensities that are intensity standardized,^32^ illustrating the challenge of low contrast amongst objects. Despite this, the ability of our DL‐R and DL‐D approach to segment the objects reasonably accurately in these dMRI sagittal slices is apparent. The green and red outlines refer to the outlines by our auto‐segmentation set‐up and expert human tracer, respectively.

For the sake of completeness, we have shown 6 slices for each organ where the GT segmentation for the organ is superimposed on the delineation generated by the proposed auto‐segmentation set‐up in Figure 7. In Figure 8, we have shown 3D renderings of the prediction results of DL‐D for the left lung, right lung, left kidney, right kidney, liver, and spleen for two subjects, along with the 3D renderings of the corresponding GT segmentations of the organs. The brightness and contrast of the display of the images in Figure 7 have been adjusted with the Microsoft Word document processing software in which this article has been written. These same images in the second column of Figure 7, with their original visual rendering based on IS^32^ are shown in Figure 9. The images in Figure 9 illustrate the insufficient clarity with which the objects are differentiated among themselves in the dMRI sagittal slices. These drawbacks with respect to intensity values could be due to the inherent tissue characteristics of the objects with respect to strong magnetic fields or due to the blurriness caused by the dynamic respiratory motion of the subject. Figure 9 demonstrates that despite these challenges, we could still achieve reasonably accurate segmentations in our medical application of interest, since ABCNet is a deep architecture with a relatively smaller number of parameters compared to other dense networks in the literature, as outlined in the second paragraph of section 3.2. The deep architecture allows for the utilization of local and global information in the pixels of the image to its advantage, while the smaller number of parameters allows the network to show itself to be generalizable.

## 5 DISCUSSION AND CONCLUSIONS

We noticed that including a seen image of a subject X at a respiratory phase “K” in training the segmentation model improves the performance of segmentation in an unseen image of X at a respiratory phase “L”. This situation is relevant to our application since we usually have manual segmentations of objects in an image of a subject at one of several respiratory phases. We can use this image to train our model, which can then be used to create auto‐segmentations of objects in the remaining respiratory phases. These can later be refined to create GT segmentations quickly for these objects in the remaining respiratory phases of interest. These additional GT segmentations in several thousand 3D images could be useful for future work toward understanding the complete respiratory dynamics in TIS compared to those in normal subjects, not just for studying the changes from EE to EI as currently practiced.

The results of Exp. 1 (shown in Table 4) and the results of the last column of Table 2 are both obtained using images at EI in the training set as well as in the testing set. The only difference between these two experiments is that the former (Exp. 1) utilized 189 subjects (∼100 images for training and validation) for evaluation, while the latter utilized 95 subjects (∼80 images for training and validation) for evaluation.

We notice that these two results are statistically similar. This observation suggests that at about 100 studies, the performance of DL‐R and DL‐D perhaps stabilizes.

From the results in Tables 4 and A2, we find that our proposed approach performs better by about 2% to 10% compared to.^15^ As mentioned earlier, the approach of^15^ is based on a 2D U‐Net architecture, whereas our architecture (ABCNet^21^) is based on an enhanced version of an encoder‐decoder architecture. We think that a judiciously designed sophisticated architecture, including DL‐R and DL‐D modules, can handle the challenges in segmentation from dMRI images better than networks such as in,^15^ since the setup works better on unseen images.

We reiterate that Mask R‐CNN is conceptually similar to our approach, except that the localization and delineation components of Mask R‐CNN are trained in a coupled manner, whereas in our proposal, the localization and delineation components are trained in a decoupled format. Table A3 shows that Mask R‐CNN has inferior performance compared to the proposal in Experiment 1 (*i.e*., on the same training and test set of Experiment 9) for 5 out of 6 organs in terms of Dice coefficient. Specifically, the location error in the z‐direction is very high for mask R‐CNN, because mask R‐CNN localizes the object in a slice‐by‐slice manner. This also explains why the location error in the z‐direction for mask R‐CNN is the least for the liver, since the liver is present in almost all of the sagittal slices. Therefore, the discrepancy in the location error along the z‐direction between the GT segmentation and that of mask R‐CNN can be expected to be less for the liver compared to other organs, such as the kidneys and spleen, which are confined to a smaller subset of slices. The coupling of localization and delineation in mask R‐CNN in a way allows the performance of the latter approach (delineation) to be affected by the imperfections of the former approach (localization). This explains why the Dice coefficient for the spleen for mask R‐CNN is low, given that the location error for the spleen is high. In our proposal, these two approaches (localization and delineation) are decoupled, making this system more robust to imperfections in the former step of localization to some extent.

Alternatively, similar to the role of the first 3D U‐Net of nn U‐Net, we might have tried segmenting the object in a down‐sampled low‐resolution image using a simple encoder‐decoder architecture, and then take the BB around this segmentation as localization information for segmenting the object in the original high‐resolution image. This idea can be explored as future work.

The evaluations shown in this paper are based on a dMRI dataset from a single center. Gathering additional datasets from multiple clinical centers might help us assess the robustness of the proposed approach. We believe that merging natural intelligence techniques with artificial intelligence (AI) techniques can have the potential to provide better segmentation performance and generalizability.^20^, ^43^ We will investigate the design of the hybrid intelligence framework for application to dMRI in patients with TIS in future work.

In this paper, we have developed an auto‐segmentation setup for the delineation of the thoraco‐abdominal organs in dynamic magnetic resonance imaging (dMRI) images of pediatric subjects. We have implemented the segmentation method in two steps: a recognition step and a delineation step. The recognition step is reasonably able to localize the organs of interest, noting that dMRI images are challenging to handle compared to other imaging modalities, especially in the pediatric setting. For the delineation of the organs, we compared two AI approaches: ABCNet^21^ and U‐Net.^15^ The delineation results for the lungs, kidneys, liver, and spleen by ABCNet from dMRI sagittal image acquisitions of the thoraco‐abdominal region of (near‐)normal healthy subjects are excellent, considering the extreme difficulty of segmentation of these objects in dMRI images. A major contribution of this paper is the conduct of multiple experiments extensively with respect to different scenarios of training and testing that can occur in practice. This is realized considering the different respiratory phases of images in the training and testing sets and the subjects to which these images belong. We are further investigating this system for segmentation of the thoraco‐abdominal organs in dMRI images of patients with thoracic insufficiency syndrome and other thoracic deformities, such as those presented in adolescent idiopathic scoliosis and early onset scoliosis.

## CONFLICT OF INTEREST STATEMENT

The authors declare no conflict of interest.

## Supporting information



Supporting information

## Data Availability

Dynamic MRI and ground truth segmentations of objects are shared via a public repository.^44^

## 1 Exp. 7: Experiment on repeated scans of the same thoraco‐abdominal region of the same subject

We reiterate that different dMRI scans, even for the same body region and for the same subject, need not have the same meaning of gray‐level intensities for an organ.^32^ Intensity standardization (IS) was developed to circumvent this problem.^32^ Of course, the manner in which the object is digitized 4‐dimensionally also changes. In this experiment, we tested whether our segmentation model can perform consistently in multiple dMRI scans of the same thoraco‐abdominal region of a subject in repeated scans. For the left lung, we obtained 2 repeated dMRI scans of each of 5 subjects with a 5‐minute gap between the two repeated scans and created auto‐segmentations of the left lung in these 20 = 5 (subjects) × 2 (EE and EI) × 2 (full scan and half scan) for the left lung. The process was repeated for the right lung with a different set of 5 subjects. The mean and SD of DC values over the 5 scans are summarized in Table A1, as well as the *p‐*values comparing DCs between the two repeated scans, which were computed based on a paired two‐tailed *t*‐testing.

**TABLE A1:** Performance of DL‐D on repeated scans in Exp.7. For details refer to subsection A.1.

Organ	1st scan	2nd scan	*p*‐value
Left lung	0.948 ± 0.016	0.900 ± 0.057	0.078
Right lung	0.919 ± 0.064	0.923 ± 0.034	0.764

We observe that the performance of DL‐D is similar in the two scans for the right lung (*p *= 0.764 based on *DC*) and the left lung (*p *= 0.078 based on DC). These results suggest that our segmentation setup can perform consistently in multiple dMRI acquisitions of the same thoraco‐abdominal region of a subject in most cases, despite the acquisitions having different intensity meanings and 4D digitization for an organ.

## 2 Exp. 8 and Exp. 9: Comparison with competing approaches

Given that articles^15^, ^16^ deal with the segmentation of the lungs only, in this experiment, we compared our work with their methods for segmentation for just the two lungs.

We took the trained model of^15^ as is for testing. That model was trained using images of 36 subjects at EE and tested on 21 subjects. Of these 36 subjects, 29 belonged to the set of 99 subjects. In Table A2, we therefore show results (*DC* and mean‐*HD*) of the method in ^15^ on images of the 70 (= 99‐29) subjects at EI and EE (Exp. 8).

**TABLE A2:** Performance of a competing approach^15^ for segmentation of left lung and right lung in terms of Dice coefficient (*DC*) and mean Hausdorff distance (mean‐*HD*) in mm in Exp.8.

Respiratory phase	Measure	Left lung	Right lung
EI	*DC*	0.833 ± 0.046	0.847 ± 0.040
	Mean‐*HD*	2.62 ± 1.22	2.94 ± 2.20
EE	*DC*	0.881 ± 0.098	0.910 ± 0.051
	Mean‐*HD*	2.23 ± 2.33	2.21 ± 2.78

Article ^16^ reports a Dice coefficient of 0.94‐0.96 for the segmentation of the lungs in dMRI using atlas‐based registration methods. The dataset that they utilized^16^ is not the same as ours with regards to MRI acquisition parameters, spatial resolution (2.81 × 2.81 × 4 mm^3^), scanner, and mode of image acquisition (regulated breathing as opposed to free‐breathing). The ages of the patients were not disclosed in.^16^ For these reasons,^16^ cannot be compared meaningfully with our method.

We also evaluated mask R‐CNN on the training and testing sets of Experiment 1. This experiment is referred to as Exp. 9. The corresponding location error in the x‐y plane and in the z‐direction is shown in the second and third rows, respectively, of Table A3 for the left lung, right lung, left kidney, right kidney, liver, and spleen. The related average and standard deviation of the Dice coefficients are shown in the fourth row of Table A3.

**TABLE A3:** Exp. 9 mean ± standard deviation of location error (*LE*) and Dice coefficient (*DC*) for mask R‐CNN for the datasets in Exp. 1.

Organ	Left lung	Right lung	Left kidney	Right kidney	Liver	Spleen
*LE* (x‐y) (mm)	7.03 ± 4.21	15.25 ± 9.53	9.09 ± 5.05	15.01 ± 9.12	4.65 ± 2.28	17.76 ± 7.56
*LE* (z) (mm)	61.00 ± 6.41	34.03 ± 25.45	68.10 ± 13.31	77.50 ± 19.57	27.80 ± 14.37	81.93 ± 12.10
DC	0.909 ± 0.018	0.909 ± 0.012	0.819 ± 0.037	0.734 ± 0.075	0.827 ± 0.036	0.350 ± 0.056
