# Supplementary material for: Auto‐segmentation of thoraco‐abdominal organs in pediatric dynamic MRI
Source: Med Phys. 2025 Nov 8;52(11):e70104. doi: 10.1002/mp.70104 (PMC12596233; doi:10.1002/mp.70104)
Supplement: Supplementary file 1 — Supporting information [file MP-52-0-s001.pdf]

SUPPLEMENTARY MATERIAL FOR
Auto-segmentation of thoraco-abdominal organs in pediatric dynamic MRI

Yusuf Akhtar[a,d], Jayaram K. Udupa[a], Yubing Tong[a], Tiange Liu[b], Caiyun Wu[a], Rachel Kogan[a], Mostafa Al-noury[a], Mahdie Hosseini[a], Leihui Tong[e], Samarth Mannikeri[a], Dewey Odhner[a], Joseph M. Mcdonough[c], Carina Lott[c], Abigail Clark[c], Patrick J. Cahill[c], Jason B. Anari[c], Drew A. Torigian[a]

[a]Medical Image Processing Group, 602 Goddard building, 3710 Hamilton Walk, Department of Radiology, University of Pennsylvania, Philadelphia, PA, 19104, USA

[b]School of Intelligence Science and Technology, University of Science and Technology Beijing, Beijing 100083, China

[c]The Wyss/Campbell Center for Thoracic Insufficiency Syndrome, Children's Hospital of Philadelphia, Philadelphia, Pennsylvania, 19104, USA

[d]School of Computer Science Engineering and Information Systems, Vellore Institute of Technology, Katpadi, Vellore-632014, Tamil Nadu, India

[e]Boston University College of Engineering, Boston University, Boston, 02215, USA

Table SUPP1*: Performance of deep learning-delineation (DL-D) in terms of Dice coefficients (mean±SD) mean-HD (mm) for Experiment 4. The 90 test images have been categorized in the first column based on the number of intermediate respiratory phases between end expiration (EE) and end inspiration (EI). For further details, please refer subsection 4.3.*

No. Int. Resp. Phases	Left lung	Right lung	Left kidney	Right kidney	Liver	Spleen	Thoraco-abdominal skin
'1'	0.66 ±0.46	0.81 ±0.74	1.17 ±0.47	1.17 ±1.60	2.07 ±2.72	**1.42 ±0.74**	2.15 ±1.15
'2'	0.70 ±0.76	0.83 ±1.02	1.62 ±1.40	1.93 ±3.15	2.17 ±1.79	1.54 ±1.15	**1.59 ±0.82**
'3'	**0.58 ±0.26**	**0.80 ±0.22**	**1.57 ±0.85**	**0.89 ±0.29**	**1.76 ±0.43**	1.65 ±0.56	1.99 ±1.18
Overall	0.68	0.82	1.98	1.64	2.12	1.51	1.79

	±0.65	±0.91	±3.75	±2.68	±2.09	±1.01	±0.98

Table SUPP2*: Performance of deep learning delineation (DL-D) in terms of (mean±SD) mean-HD (mm) for Experiment 5. The 90 test images have been categorized in the first column based on the number of intermediate respiratory phases between end expiration (EE) and end inspiration (EI). For further details, please refer subsection 4.3.*

No. Int. Resp. Phases	Left lung	Right lung	Left kidney	Right kidney	Liver	Spleen	Thoraco-abdominal skin
'1'	0.51 ±0.23	0.76 ±0.63	1.48 ±2.07	1.23 ±1.49	2.13 ±2.95	1.80 ±1.54	2.83 ±1.62
'2'	0.54 ±0.23	0.97 ±1.43	1.29 ±0.83	1.45 ±1.68	2.18 ±1.96	**1.37 ±0.63**	2.08 ±1.08
'3'	**0.39 ±0.17**	**0.55 ±0.31**	**0.89 ±0.23**	**0.81 ±0.38**	**1.50 ±0.63**	1.40 ±0.43	**1.49 ±0.96**
Overall	0.52 ±0.23	1.12 ±2.56	1.33 ±1.34	1.57 ±2.62	2.13 ±2.27	1.51 ±1.01	2.29 ±1.43

Table SUPP3*: Performance of deep learning delineation (DL-D) in terms of (mean±SD) mean-HD (mm) for Experiment 6. The 99 test images have been categorized in the first column based on the number of intermediate respiratory phases between end expiration (EE) and end inspiration (EI). The best performing case amongst '0', '1', '2', and '3' intermediate respiratory phases has been highlighted. For further details, please refer subsection 4.3.*

No. Int. Resp. Phases	Left lung	Right lung	Left kidney	Right kidney	Liver	Spleen	Thoraco-abdominal skin
'0'	**0.43 ±0.21**	0.78 ±0.46	2.32 ±2.29	**1.03 ±0.62**	1.79 ±1.22	1.77 ±1.99	2.45 ±0.98
'1'	0.79 ±1.56	1.86 ±1.27	6.83 ±20.67	1.29 ±1.55	1.74 ±2.62	2.00 ±3.50	2.53 ±1.15
'2'	0.72 ±2.31	0.76 ±1.29	1.70 ±3.79	3.15 ±10.26	2.35 ±3.89	2.29 ±5.17	2.45 ±1.67
'3'	**0.43 ±0.21**	**0.37 ±0.12**	**1.35 ±0.72**	1.12 ±0.54	**1.74 ±0.91**	**1.60 ±0.65**	**2.26 ±0.81**

Overall	0.69 ±0.21	1.04 ±0.12	3.16 ±0.72	2.28 ±0.54	2.08 ±0.91	2.11 ±0.65	2.46 ±0.81
